# Supplementary material for: Trends in suicide deaths before and after the COVID-19 outbreak in Korea
Source: PLoS One. 2022 Sep 12;17(9):e0273637. doi: 10.1371/journal.pone.0273637 (PMC9467344; doi:10.1371/journal.pone.0273637)
Supplement: S1 Table — (DOCX) [file pone.0273637.s001.docx]

| S1 Table. Performance metrics for forecasting models. | | | |
| --- | --- | --- | --- |
| Model | RMSE | MAE | MAPE |
| Total | | | |
| Prophet  (changepoint.prior.scale = 0.02) | 88.13 | 66.88 | 5.86 |
| ARIMA  (1,0,2)(1,1,2)[12] | 128.11 | 112.67 | 9.91 |
| Men | | | |
| Prophet  (changepoint.prior.scale = 0.04) | 59.81 | 44.28 | 5.47 |
| ARIMA  (2,0,2)(1,1,0)[12] | 48.05 | 43.58 | 5.41 |
| Women | | | |
| Prophet  (changepoint.prior.scale = 0.01) | 36.59 | 29.71 | 8.86 |
| ARIMA  (0,1,2)(1,1,1)[12] | 58.25 | 44.66 | 12.43 |
| Age ≤ 34 | | | |
| Prophet  (changepoint.prior.scale = 0.03) | 25.13 | 19.44 | 9.72 |
| ARIMA  (2,1,1)(0,0,2)[12] | 35.05 | 27.60 | 12.99 |
| Age 35–49 | | | |
| Prophet  (changepoint.prior.scale = 0.05) | 38.75 | 32.37 | 10.39 |
| ARIMA  (1,1,1)(2,0,0)[12] | 32.10 | 28.13 | 9.14 |
| Age 50–64 | | | |
| Prophet  (changepoint.prior.scale = 0.02) | 25.49 | 19.68 | 5.83 |
| ARIMA  (1,1,1)(1,1,2)[12] | 19.15 | 16.36 | 4.97 |
| Age ≥ 65 | | | |
| Prophet  (changepoint.prior.scale = 0.02) | 21.42 | 16.64 | 5.76 |
| ARIMA  (3,1,2)(2,1,0)[12] | 19.15 | 16.36 | 4.97 |
| Abbreviations: RMSE, Root Mean Squared Error; MAE, Mean Absolute Error; MAPE, Mean Absolute Percentage Error; ARIMA, Autoregressive Integrated Moving Average. | | | |
