# Supplementary figures and images for: Trends in suicide deaths before and after the COVID-19 outbreak in Korea
Source: PLoS One. 2022 Sep 12;17(9):e0273637. doi: 10.1371/journal.pone.0273637 (PMC9467344; doi:10.1371/journal.pone.0273637)

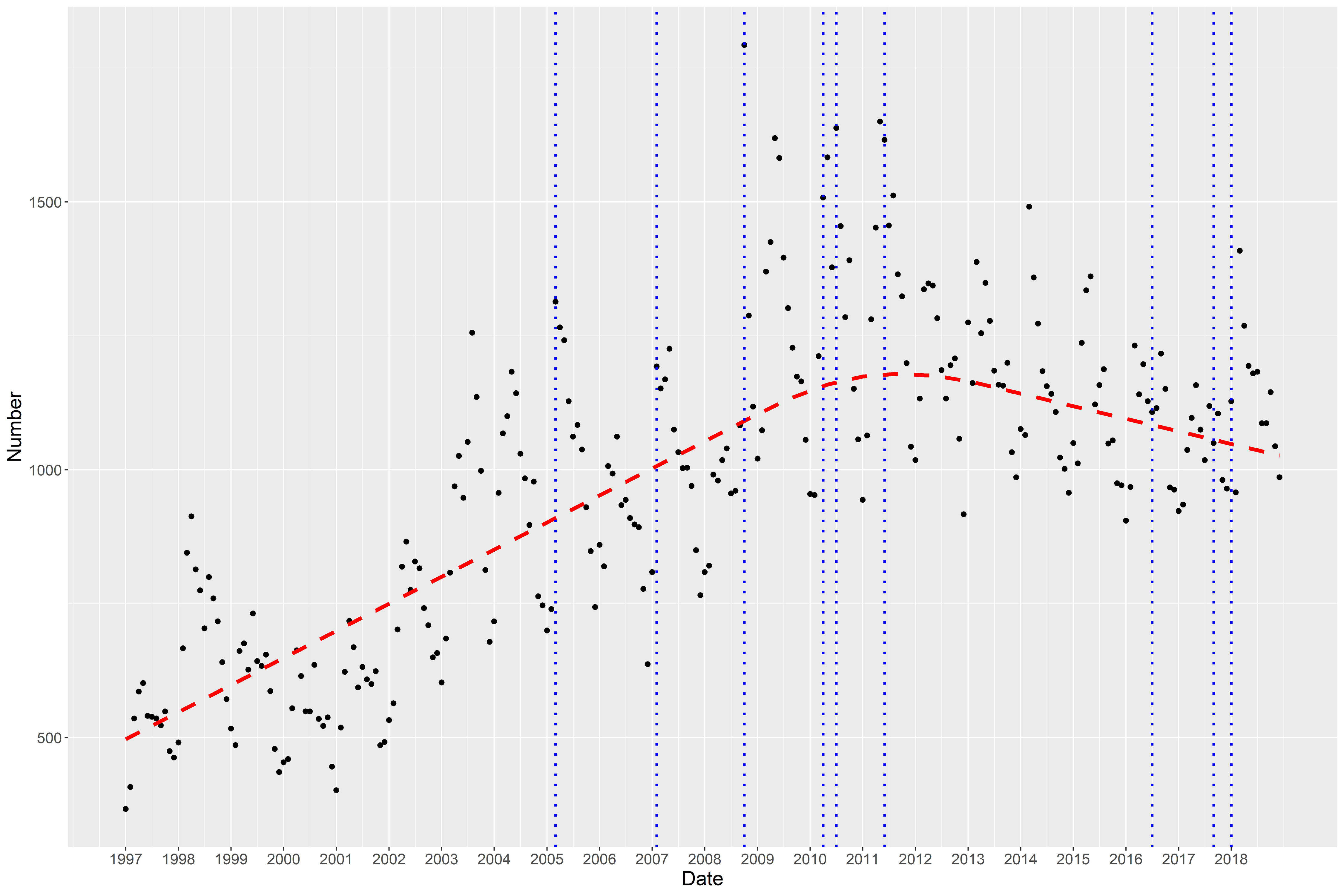

Supplement: S1 Fig — The dots represent the numbers of suicides and the red dashed line indicates the trend. The blue vertical dotted lines indicate the dates of celebrity suicides. (TIF) [file pone.0273637.s002.tif]

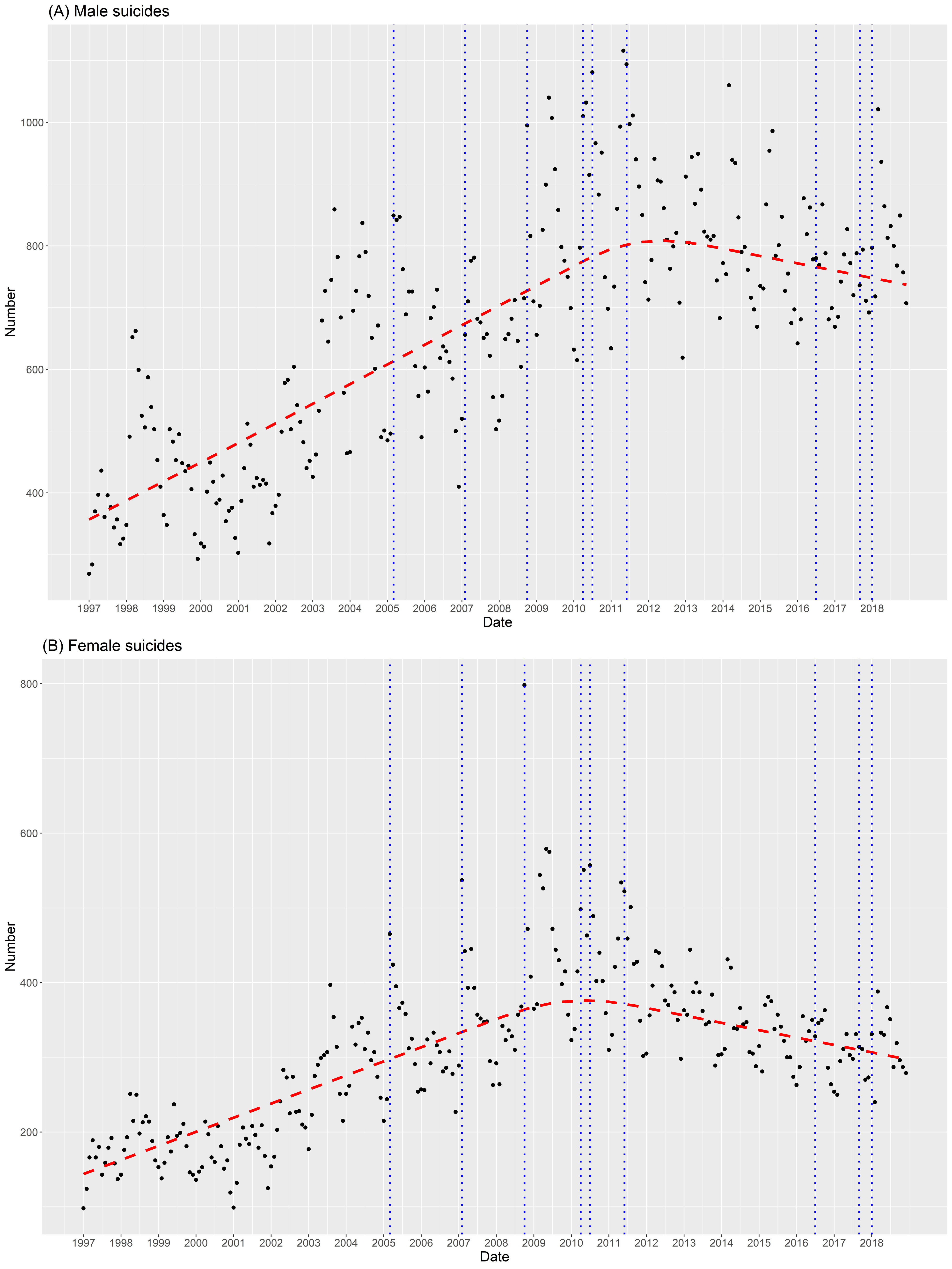

Supplement: S2 Fig — The dots represent the numbers of suicides and the red dashed line indicates the trend. The blue vertical dotted lines indicate the dates of celebrity suicides. (TIF) [file pone.0273637.s003.tif]

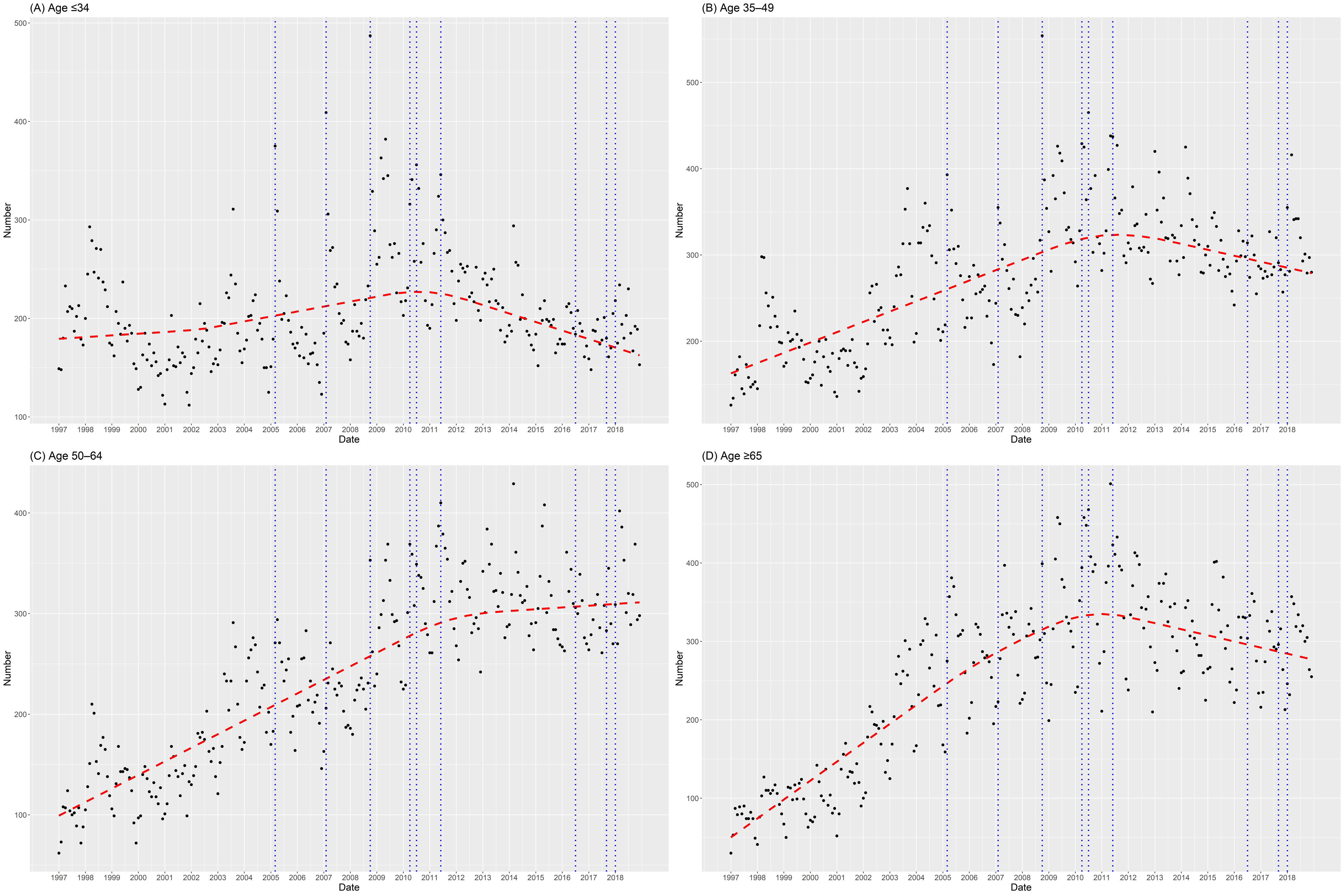

Supplement: S3 Fig — The dots represent the numbers of suicides and the red dashed line indicates the trend. The blue vertical dotted lines indicate the dates of celebrity suicides. (TIF) [file pone.0273637.s004.tif]
