# Supplementary material for: Trends in suicide deaths before and after the COVID-19 outbreak in Korea
Source: PLoS One. 2022 Sep 12;17(9):e0273637. doi: 10.1371/journal.pone.0273637 (PMC9467344; doi:10.1371/journal.pone.0273637)
Supplement: Attachment — Submitted filename: Response to reviewers.docx [file pone.0273637.s006.docx]

**Response to the Reviewers' comments:**

Reviewer #3: This is, in summary, an interesting study aimed to investigate the effect of the coronavirus disease-2019 (COVID-19) pandemic on suicide trends in Korea via a time-series analysis. The authors found that the total numbers of suicides during the COVID-19 pandemic did not deviate from projections based on the pre-pandemic period. In addition, the number of suicides among females and those under the age of 34 years significantly exceeded the expected level. Moreover, the COVID-19 pandemic did not increase the overall suicide rate significantly. Finally, suicides among women and young people increased, suggesting that the pandemic might drive more members of these groups to suicide

Overall, the present manuscript is interesting and well-written in its current version; thus, only minor changes are required, in my opinion.

**- Response: We thank Reviewer #3 for the thoughtful suggestions.**

First, when throughout the Introduction section, the authors correctly stressed the psychosocial impairment and disability in the context of COVID-19, they might further stress the impact of Covid-19 related lockdown on lifestyle habits and behavioral risk factors. Importantly, the impact of COVID-19 lockdown on physical, mental, and social wellbeing of elderly and fragile populations in specific countries such as Italy cannot be ignored. The multi-disciplinary competencies together with appropriate funding and access to rich data sources may allow to fulfill interesting research objectives. Thus, according to this background, the study of Odone and coworkers published on Acta Biomed (PMID: 32701921) may be cited within the main text.

**- Response: Following the reviewer’s comments, we have cited the Odone et al.’s study (PMID: 32701921) in the Introduction section (highlighted, page 3 line 9 – 10).**

In addition, the authors might further mention, in the context of Covid-19 infection, the link between the deterioration of people’s mental health and the consequent increase in suicide rate which is frequently underreported. Unfortunately, above 2% of the traffic accidents are suicide behaviors. This phenomenon may be underreported considering that suicides by car accidents may be reported as accidental in the national statistics. Therefore, given the above information, my suggestion is to include within the manuscript, the study published in 2016 on Forensic Sci Int (PMID: 22576104).

**- Response: Thanks for the reviewer’s comments. We have added the reference (PMID: 22576104) in the Limitation section (highlighted, page 14 line 223 – 224).**
